# A novel implementation of mARC treatment for non-dedicated planning systems using converted IMRT plans

**DOI:** 10.1186/1748-717X-8-193

**Published:** 2013-08-03

**Authors:** Yvonne Dzierma, Frank Nuesken, Norbert Licht, Christian Ruebe

**Affiliations:** 1 Department of Radiation Oncology, Saarland University Medical Centre, Kirrberger Str. Geb. 6.5, D-66421 Homburg, Saarland, Germany

**Keywords:** Modulated arc (mARC), Siemens Artiste, IMRT to arc conversion, Beam angle optimization

## Abstract

**Background:**

The modulated arc (mARC) technique has recently been introduced by Siemens as an analogue to VMAT treatment. However, up to now only one certified treatment planning system supports mARC planning. We therefore present a conversion algorithm capable of converting IMRT plans created by any treatment planning system into mARC plans, with the hope of expanding the availability of mARC to a larger range of clinical users and researchers. As additional advantages, our implementation offers improved functionality for planning hybrid arcs and provides an equivalent step-and-shoot plan for each mARC plan, which can be used as a back-up concept in institutions where only one linac is equipped with mARC.

**Methods:**

We present a feasibility study to outline a practical implementation of mARC plan conversion using Philips Pinnacle and Prowess Panther. We present examples for three different kinds of prostate and head-and-neck plans, for 6 MV and flattening-filter-free (FFF) 7 MV photon energies, which are dosimetrically verified.

**Results:**

It is generally more difficult to create good quality IMRT plans in Pinnacle using a large number of beams and few segments. We present different ways of optimization as examples. By careful choosing the beam and segment arrangement and inversion objectives, we achieve plan qualities similar to our usual IMRT plans. The conversion of the plans to mARC format yields functional plans, which can be irradiated without incidences. Absolute dosimetric verification of both the step-and-shoot and mARC plans by point dose measurements showed deviations below 5% local dose, mARC plans deviated from step-and-shoot plans by no more than 1%. The agreement between GafChromic film measurements of planar dose before and after mARC conversion is excellent. The comparison of the 3D dose distribution measured by PTW Octavius 729 2D-Array with the step-and-shoot plans and with the TPS is well above the pass criteria of 90% of the points falling within 5% local dose and 3 mm distance to agreement. For all plans, the treatment time was noticeably reduced by conversion to mARC.

**Conclusions:**

We present the feasibility test for converting IMRT step-and-shoot plans from the RTP-output of any treatment planning system (Philips Pinnacle and Prowess Panther, in our case) into mARC plans. The feasibility and dosimetric equivalence is demonstrated for the examples of a prostate and a head-and-neck patient.

## Background

The modulated arc (mARC) technique [1,2] has recently become available for Siemens Artiste linear accelerators as an analogue to RapidArc and volume modulated arc therapy (VMAT) available for Varian and Elekta machines ([3]; for a recent review see [4]). Both techniques offer highly conformal treatment, since inversion is performed similarly to intensity-modulated radiotherapy (IMRT) for a large number of beam directions, which may create a complete or partial gantry rotation; a notable decrease in treatment time is accomplished by the continuous gantry rotation and multi-leaf collimator (MLC) movement. However, mARC differs from VMAT in underlying philosophy and practical implementation. Whereas for VMAT, the beam is kept on during the complete arc, while the MLC, collimator and gantry move to reach predetermined optimized configurations at the control points, the mARC only switches the beam on inside short “arclets“ around the optimization points. Between the “control points“ that define the start and end of an arclet, the MLC and collimator configuration are kept fixed; the width of the arclet is chosen as small as possible from the point of view of monitor units (MU) and gantry rotation velocity, while always remaining within user-defined upper limits. This method offers an additional degree of freedom in treatment planning in the sense that the treatment planning system (TPS) is not confined in its choice of MLC pattern and collimator by the gantry rotation velocity; rather, the gantry can be stopped between arclets until the desired configuration is reached. This expands the solution space of the optimization; at the same time, it may be argued that dosimetric agreement between the treatment plan and the delivered dose is improved, since intermediate MLC/collimator configurations are excluded.

At the time of writing, only the Prowess Panther TPS supports mARC plannning; the certification for RayStation is pending. The present study presents a novel implementation of mARC planning based on any kind of TPS capable of IMRT planning and RTP export. For clinics relying on treatment planning systems other than Prowess/RayStation, our conversion algorithm offers the possibility of implementing mARC without switching to Prowess/RayStation, within the familiar environment of their own TPS. We hope that this will make the excellent functionality of mARC available to a much larger range of clinical users and researchers. In the clinical context, this brings Siemens users to the point of arc treatments which have hitherto been missing; in research, the different approach to arc planning merits further research, since a comparison with VMAT and RapidArc may be interesting for future developments in this field. As an additional advantage, our conversion method provides an equivalent step-and-shoot plan for each mARC plan, which can be used as a back-up concept in institutions where only one linac is equipped with mARC.

Furthermore, the implementation presented offers improved functionality for the planning of hybrid arcs (compare [5]), in which the arc is complemented by one or a number of static fields with several segments, similar to IMRT beams. In contrast to hybrid planning in existing TPS, our approach offers the possibility of planning in hybrid mode without *a priori* choosing the static gantry angles; instead, the optimum hybrid beam directions are determined by the inversion algorithm.

This work is meant as a feasibility study to outline a practical implementation of mARC planning using Philips Pinnacle as an example, with an outlook on the possibilities for further research that can be based on this modality. As a basis to the demonstration of feasibility, we show and discuss some examples of how plans may be created and converted, but without the intention of providing a complete planning study. The same conversion code was also tested for conversion of Prowess Panther IMRT plans to ensure that the algorithm works independently of the original planning system. To our knowledge, this is the first proof-of-principle of converting an IMRT treatment plan into an equivalent single/hybrid arc.

## Method

### Technical implementation

The Department of Radiotherapy of the Saarland University Medical Centre is equipped with three linacs (two Siemens Artiste, one Siemens Oncor) with 160 MLC, out of which one Artiste with 6 MV and flattening-filter-free (FFF) 7 MV photon energies received the mARC upgrade. The 6 MV energy is matched with the other two linacs, which do not yet support mARC treatment. CT-based (Philips BigBore) treatment planning is routinely performed using the Philips Pinnacle TPS (V9.2 and V9.4); for the mARC, Prowess Panther was additionally commissioned. Treatment plans are exported from Pinnacle via DICOM and loaded into Mosaiq (Elekta), then transferred to Syngo (Siemens) and sent to the linac for treatment.

Treatment planning for mARC is generally performed along the same lines as “standard“ IMRT treatment planning, the main difference being that the optimization points along an arc are placed equidistantly (generally at 5°-10° intervals), with one “segment“ (MLC/collimator configuration) intended for each gantry direction.

The user defines the maximum arclet length over which the dose for each optimization point may be spread; the Artiste firmware will restrict this angle as much as technically feasible, but never exceed it. As the TPS only calculates the dose at the optimization points, the delivered dose will deviate from the calculated dose slightly, which is why long arclets (5° or more) should be excluded.

In mARC planning, the user may decide beforehand to favour particular beam directions for which several segments are allowed, resulting in a hybrid arc. For Pinnacle IMRT plans, the creation of multiple-segment beams can be permitted by setting the maximum number of segments larger than the number of beams. In contrast to mARC, this will let the TPS decide for which gantry direction these segments will be applied. The optimization will therefore automatically choose the directions of hybrid beams (if any), hence providing an additional degree of freedom in comparison with hybrid mARC planning.

**Figure 1:**
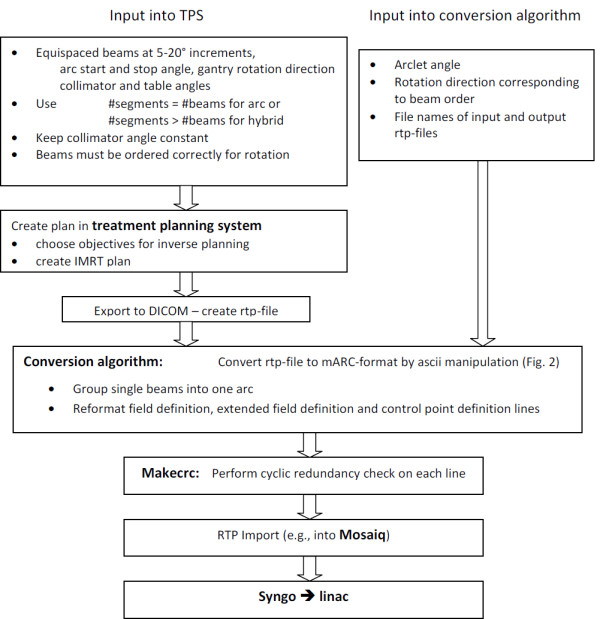
Schematic workflow of treatment plan creation, conversion into mARC until transfer to the linear accelerator.

Our approach to mARC planning in Pinnacle relies on performing “normal“ IMRT planning using direct machine parameter optimization (DMPO) for 18 to 36 beams, and then reformatting the output RTP-file [6] to conform to mARC standards (Figure 1). In principle, this is the same method as is applied in SmartArc planning, where a first IMRT-like optimization is performed using beams spaced between 12° and 24° apart, the segments of which are then reorganized to create an arc with control points every 2° to 6° [7]. Therefore, each RapidArc plan can be interpreted as an equal-quality IMRT plan with one beam and one segment placed at each control point (resulting in a larger number of beam directions than routinely used in IMRT treatments). Vice versa, we move from the IMRT planning with equidistantly spaced beams, possibly including more than one segment, to an arc treatment to be irradiated as mARC. This method combines the advantage of arc-based treatment, which is both the speed and the many gantry angles, with the advantage of IMRT, i.e. free choice of MLC configuration and optimal distribution of hybrid segments.

Formally, the RTP-file output of a hybrid arc plan is similar to the output of an IMRT plan, with the main difference that the Pinnacle IMRT plan interprets each gantry direction as a separate beam with an arbitrary number of segments, whereas a hybrid arc is interpreted as a single beam. Consequently, the coding of the field and control points differs somewhat between the two output formats (mARC vs. step-and-shoot). In particular, the Pinnacle export RTP-files of single-segment beams in IMRT plans have only one control point rather than two (at the beginning and end of the segment) for mARC, and the dose is normalized for each beam rather than for a complete arc. Several minor pieces of information in the field and control point definitions also require adjustments (gantry rotation direction from one arclet to the next, field size, dose rate etc.). The task of our conversion algorithm is to perform all these input and reformatting adjustments in a way to create a new RTP-file which will look like an mARC plan and can be imported for treatment.

**Figure 2:**
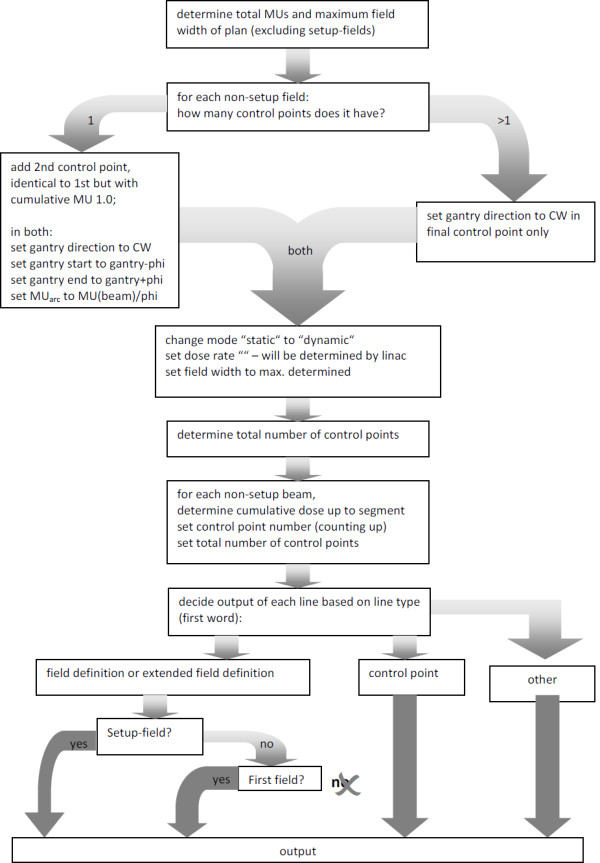
Principle of mARC conversion algorithm.

Since the RTP-file can be opened with any text editor, reformatting can be performed automatically: we used *tcsh*-scripting with *awk*, but any kind of ASCII file manipulation code will serve the purpose. The main steps of the algorithm are summarized in Figure 2.

After importing the modified RTP-file into Mosaiq, the remaining part of the workflow remains as before. If it is so desired, the original plan can be imported as an alternative back-up concept, which would dosimetrically produce the same plan, but be interpreted as a step-and-shoot IMRT with correspondingly longer treatment time.

### Planning examples

We present planning examples for two cases routinely treated with IMRT at our institutions: a prostate patient and a head-and-neck patient, which are both planned using three different methods for the two available energies: flat 6 MV and FFF 7 MV, respectively. Since this is meant only as a proof of principle and not as a planning study, the main focus of our paper is on the feasibility of converting the plans into mARC; the decision exactly how the original step-and-shoot plans are created is left to the user depending on the treatment planning system used and on the clinical requirements. However, we believe it is of interest to show different ways in which planning can proceed using the Pinnacle TPS, and the resulting quality of the plans, compared with “normal” IMRT plans irradiated at our institution (Table 1). We limit ourselves to PTV plans, since there is more variability in the boost depending on the tumour location; the boost planning and conversion can be performed along the same lines as shown for the PTV.

**Table 1:** Overview of planning and conversion scenarios

	**Original step-and-shoot plan**	**Conversion**	**Converted mARC plan**
Prostate			
Version a	19 beams	Convert directly as is	8 arclets
	35 segments		11 hybrid fields
Version b	19 beams 35 segments Same plan as version a	Split beams with more than 1 segment into two beams spaced 4° apart (one beam −2°, one +2° relative to original), this gives 26 beams, then convert	22 arclets 4 hybrid fields
Version c	30 beams 35 segments	Convert directly	25 arclets 5 hybrid fields
Head and neck			
Version a	18 beams 50 segments	Split each beam with More than 1 segment into two beams spaced 6° apart (one beam −3°, one +3° relative to original)	26 arclets 5 hybrid fields
Version b	36 beams	Convert directly	27 arclets
	50 segments		9 hybrid fields
Version c	36 beams 72 segments	Do not change beams, but order segments in a way that two rotations are made, so the first segment of each beam is irradiated on the cw arc; the rest of the segments are included in the ccw arc	Clockwise: pure mARC including only 36 arclets (not hybrid) Counter-clockwise: 23 arclets, 6 hybrid fields

For the prostate tumor, the first plan (version a) is based on an original 19 beam IMRT plan with 35 segments (1–3 segments per beam). This is directly converted into mARC, corresponding to an “extreme hybrid“ case in which over 10 beams are hybrids. This plan is still much more coarsely spaced than a normal arc plan; compared with IMRT, the converted plan already has the advantage of static jaws and arclets.

Starting from this original plan version a, manual field separation is performed to create more closely spaced beams, by separating those beams with two segments into two beams spaced 4° apart; beams with one segment or three segments are kept unchanged. After conversion of this second (version b) plan into mARC, beams with three segments appear as hybrid fields, whereas beams with one segment and the divided beams (originally with two segments) become normal arclets. This process results in a hybrid plan with a much smaller number of hybrid fields and a more “arc-like” beam configuration.

As a third alternative, a “real“ hybrid arc plan is optimized using 30 equidistant beams spaced 12° apart, again allowing 35 segments (version c), and converting the resulting plan to mARC. This plan now has only five segments which can create a maximum of five hybrid fields, whereas the rest of the beams become normal arclets. This technique gives a similar “arc-like” beam configuration to version b, but with much less manual manipulation and splitting of beams.

For the head-and-neck case, planning is more complicated since a larger number of segments is usually needed to achieve good quality IMRT plans. This is mirrored in mARC planning – using Prowess, we also find it difficult to obtain good quality head-and-neck plans using just one single arc. We therefore create a first version IMRT plan with 18 beams and 50 segments, which has more than one segment in each beam. If the conversion to mARC shall make sense, at least one single-segment beam must exist to be converted into an arclet. We therefore split all those beams with two segments into two beams spaced 6° apart – this version (a) is converted into mARC.

A second version (b) is created using 36 beams at 10° increments and 50 segments, so that conversion into mARC results in at least 22 arclets.

Head-and-neck target volumes generally require more segments for good plan quality than prostate plans, and arc treatments for these target volumes often involve two or three arcs to achieve a good dose distribution with regard to PTV coverage and sparing of organs at risk. Therefore, we performed a third inversion (version c) using 36 beam directions with 72 segments. Most beam directions now have two segments, a few have one segment or more than two. The final plan can be manually split into two rotations, one clockwise and the other counter-clockwise, which are converted separately. For this, beams with two segments can be distributed evenly to the two arcs, beams with one segment only appear in one arc, and beams with more than two segments are used to create a single-segment beam in the first arc and a hybrid field with the remainder of the segments in the second arc. The manual conversion of the two plans with different gantry rotation directions is somewhat cumbersome; however, this is intended as a proof of principle, a direct splitting and conversion to two or more arcs are envisioned for future implementation in the conversion algorithm.

For all plans, direct machine parameter optimization over 40 iterations is performed in Pinnacle using the same objectives and constraints we usually apply for IMRT. CT-based dose calculation is on a 0.2 × 0.2 × 0.2 cm^3^ dose grid with the collapsed cone algorithm. Reporting of IMRT dose distributions is based on the guidelines of the International Commission on Radiation Units and Standards [8].

The conversion algorithm works on the RTP-file-level, which is in principle independent of the planning system used to create the original IMRT plans. Nonetheless, we demonstrate the feasibility for a second choice of planning system using a Prowess Panther prostate IMRT plan with 30 beams of one segment each. The IMRT plan was optimized in Prowess Panther using our standard criteria and the dose was calculated on a 0.3 × 0.3 × 0.3 cm^3^ grid using the collapsed cone algorithm. After exporting the treatment plan, conversion and irradiation were performed in the same way as for the Pinnacle plans.

### Dosimetric verification

Each plan is converted into an mARC plan according to the conversion algorithm presented above, with a maximum arclet angle of 4°. Dosimetric verification involves comparison of the converted mARC plan with the original IMRT step-and-shoot plan and with the calculated dose from the Pinnacle TPS. Both absolute dose and fluence distribution are assessed. For the absolute dose, point dose measurements are performed in an acrylic phantom (BrainLab) with a semiflex ionization chamber (PTW type 31010) in the same way routinely performed for IMRT verification. The planar dose distributions of the step-and-shoot plan and mARC plan are compared using GafChromic film measurements inside the same acrylic phantom and PTW Octavius with 729 2D-Array, analyzed in the PTW VeriSoft software. For a comparison of the measured 3D dose distribution (both absolute dose and fluence distribution) with the calculated dose from the treatment planning system, the PTW Octavius with 729 2D-Array is used.

## Results

### Planning and plan quality

The creation of the plans in Pinnacle followed the same procedure as routinely applied for IMRT planning and was hence straightforward. Splitting the beams (version b for the prostate patient and version a for the head-and-neck example) involved some time-consuming manual changes in the plans, but without difficulties. The same applies to the head-and-neck version c, which rotates first clockwise and then counter-clockwise: again, splitting the original plan into two plans involved some manipulation of the plans, but was straightforward in principle. Two plans were then exported and separately converted into arcs, they could be irradiated in sequence without problems.

The main focus of this study is the technical feasibility of the conversion of step-and-shoot plans into mARC, disregarding how the original plans were created. We have mentioned several ways just to give an example of how one may proceed from IMRT to mARC using Pinnacle, but the possibilities vary with the treatment planning system, and our examples are neither meant to be exclusive nor offer optimum advice on how best to create good quality plans with a maximum of beam angles and a minimum of segments per beam. The creation of good step-and-shoot plans for mARC conversion is not trivial particularly for complicated target volumes such as head-and-neck tumours – in fact the up-front creation of mARC plans for these indications is complicated even when using a dedicated planning system such as Prowess. We therefore believe it is of interest to demonstrate the quality of the plans we created for our example cases, so the reader gets an impression of the outcome from the different methods. We stress again that this is not intended as a planning study and offered only as an aside, which is why in-detail analysis of quality measures (Table 2) are omitted.

**Table 2:** Quality measures of the plans presented as examples

**Prostate patient**				
**Measures of quality**		**Standard IMRT plan**	**Version a/b, 6 MV**	**Version c, 6 MV**
PTV	D_2_ (Gy)	51.73	53.98	53.59
	D_50_ (Gy)	50.60	51.62	51.45
	D_98_ (Gy)	48.13	45.59	44.97
	HI	0.07	0.16	0.17
Rectum	D_2_ (Gy)	50.60	51.05	50.55
	D_30_ (Gy)	35.18	36.25	35.63
	D_50_ (Gy)	24.94	21.61	19.93
Bladder	D_2_ (Gy)	51.28	52.69	52.63
	D_30_ (Gy)	48.97	43.06	43.12
	D_50_ (Gy)	34.50	14.69	13.57
Femoral head (right)	D_2_ (Gy)	32.99	37.43	34.50
	D_30_ (Gy)	24.09	25.39	24.37
	D_50_ (Gy)	20.49	18.46	18.13
**Head-and-neck patient**				
**Measures of quality**		**Standard IMRT plan**	**2 arcs FFF 7 MV (version c)**	
PTV	D_2_ (Gy)	54.47	53.32	
	D_50_ (Gy)	45.55	44.86	
	D_98_ (Gy)	50.55	50.67	
	HI	0.18	0.17	
Right parotid	D_2_ (Gy)	7.30	5.41	
	D_50_ (Gy)	48.37	51.82	
	D_mean_ (Gy)	15.36	13.62	
Spinal cord	D_2_ (Gy)	21.28	21.28	
	D_50_ (Gy)	25.36	25.25	
	D_mean_ (Gy)	19.77	19.30	

The values presented are based on the ICRU Report 83 [8]. The homogeneity index is defined as HI = (D_2_ – D_98_) / D_50_. We only give dose values for the right femoral head and right parotid, since they receive higher doses than their left counterparts.

**Figure 3:**
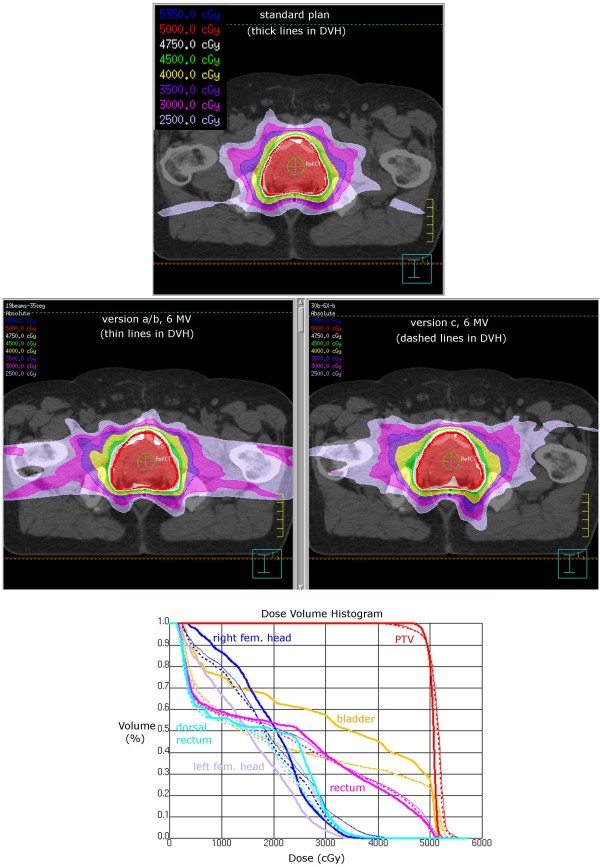
**Example dose distributions of prostate treatment plan.** A standard prostate plan (irradiated for the patient) is shown on top, the dose distributions of plan versions a/b and c (6 MV) in the middle panel, with dose volume histogram (lower panel). In the DVH, thick lines correspond to the standard IMRT plan, thin lines to prostate plan version a/b (no noteable difference between the two plans), and dashed lines to prostate plan version c.

Creation of plans with very few segments per beam is generally complicated in Pinnacle, even more so when flattening-filter-free beams are used. For the prostate patient, decent quality plans could be achieved with little difficulty using IMRT with either 19 beams or 30 beams and 35 segments; in both cases the flat 6 MV plan gave better dose distribution, so we present these two plans as compared with the clinically radiated plan for the same patient (Figure 3). The splitting of the beams did not introduce great changes in the plans, in particular the very symmetrical and conformal prostate plans. Prostate plans version a and b are virtually identical with respect to DVH and quality measures, only few slices show a slightly different dose distribution in several voxels, which is negligible. We therefore present prostate plan versions a/b combined without distinguishing between the two. All prostate 6 MV plans are acceptable according to our in-house standard in that the PTV is covered by the 95% isodose, although coverage is slightly reduced and the maximum slightly higher (i.e. homogeneity is effectively worse) than for the treated plan. The dose to the organs at risk is similar to the original plan, with a decrease in dose to the bladder for the arc plans. Depending on whether PTV homogeneity or dose to the bladder is weighted higher, the decision would be either for the arc plans or for the originally treated plan. The FFF 7 MV plans were discarded because although satisfying all the criteria (PTV coverage and dose to organs at risk), they had higher dose gradients close to the rectum, which would endanger the rectum in the case of positioning uncertainties or intra-fraction motion.

For the head-and-neck example, little difference was observed between the plan qualities of 6 MV and FFF 7 MV plans. Possibly, this may be indebted to a better choice of inversion objectives or constraints, which is equally adequate for flat and FFF beams. In fact, the inversion objectives for the head-and-neck case were taken from a planning study comparing IMRT performance of the flat and FFF beam (Dzierma et al., in prep.), which found equally good performance of both as long as the inversion objectives were modified to be suitable for both. Since no experience exists at our institution for FFF prostate IMRT planning (FFF beams are being introduced for prostate IMRT, but no standard template of objectives has yet evolved), the poorer plan quality of the FFF beam versions in the prostate example may just be caused by a less-than-optimal choice of inversion objectives.

**Figure 4:**
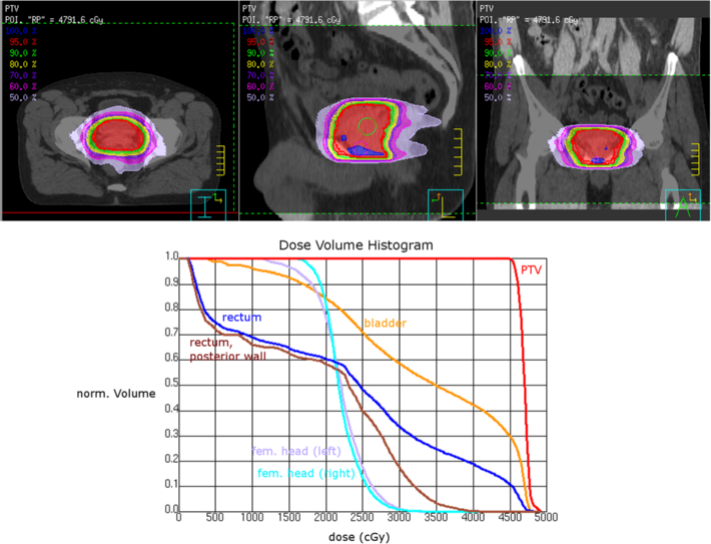
**Example dose distribution and DVH of a clincally treated prostate plan (PTV IMRT planned with 30 beams and 30 segments and converted to mARC for treatment).** Prescription was for the 95% isodose.

The prostate plans *a posteriori* created for mARC conversion have somewhat worse quality than the original treated plan. They were included here to demonstrate several possible planning procedures; however, the optimization might still be improved to achieve better PTV coverage. We show an example of a good quality plan created for clinical treatment in Figure 4.

**Figure 5:**
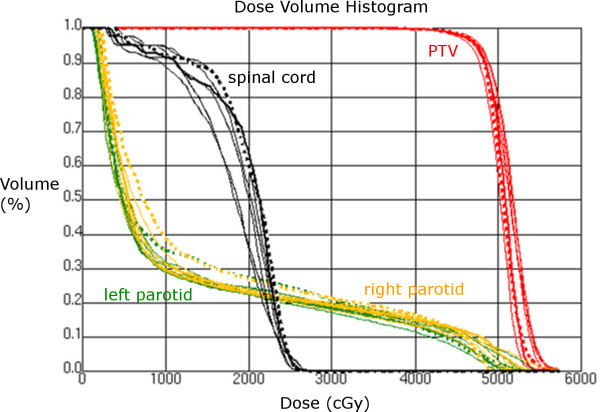
**Dose volume histogram (DVH) of head-and-neck patient plans.** The thick dashed line shows the DVH for a standard IMRT plan generally used at our institution. The thick line corresponds to the FFF 7 MV plan version c, displayed in Figure 6. Thin lines correspond to the other plan versions a,b,c (6 MV and 7 MV) – these plans are not analyzed in detail here and are therefore not distinguished in the figure.

**Figure 6:**
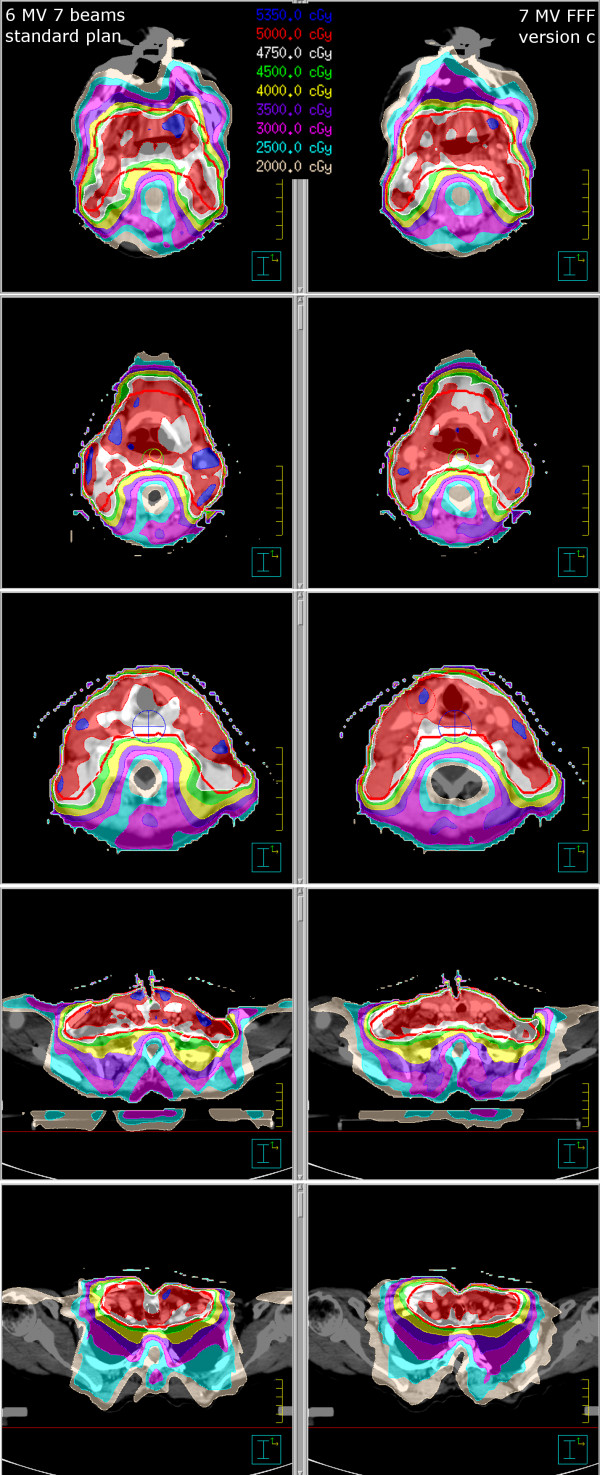
**Example dose distributions of head-and-neck treatment plan.** The left panel shows our standard IMRT plan (also displayed in Figure 5 as thick lines in the DVH), the right panel shows version c converted plan (FFF 7 MV), both in different CT slices.

In the head-and-neck case, plan versions a and c had acceptable qualities. Version b, with 50 segments distributed over 36 beam directions, gave worse quality than both version a (50 segments with 18 beam directions, giving more opportunity of modulation for each beam) and version c (36 beams with 72 segments), even though the DVH is similar for all six plans (both 6 MV and FFF 7 MV, Figure 5). Version c had best coverage and least extension of the 80% isodose outside the PTV, which is why we chose to present this plan (FFF 7 MV, which has comparable quality to the 6 MV version c plan, compared with a “standard” IMRT plan using 7 beams and 50 segments (Figure 6, Table 2)).

### Conversion and dosimetric verification

The conversion of the plans to mARC format – performed as described above – yielded functional plans with no further modifications, which could be imported and irradiated without incidences. There was no difference in handling or feasibility between IMRT plans created in Philips Pinnacle and Prowess Panther. For each plan version, we perform dosimetric testing of the original step-and-shoot plan versus the converted mARC plan.

Absolute dosimetric verification of both the step-and-shoot and mARC plans by point dose measurements showed deviations below 5% local dose, which is within the specifications allowed at our institution for IMRT verification. The absolute dosimetric verification of mARC plans deviated from step-and-shoot plans by no more than 1%.

**Figure 7:**
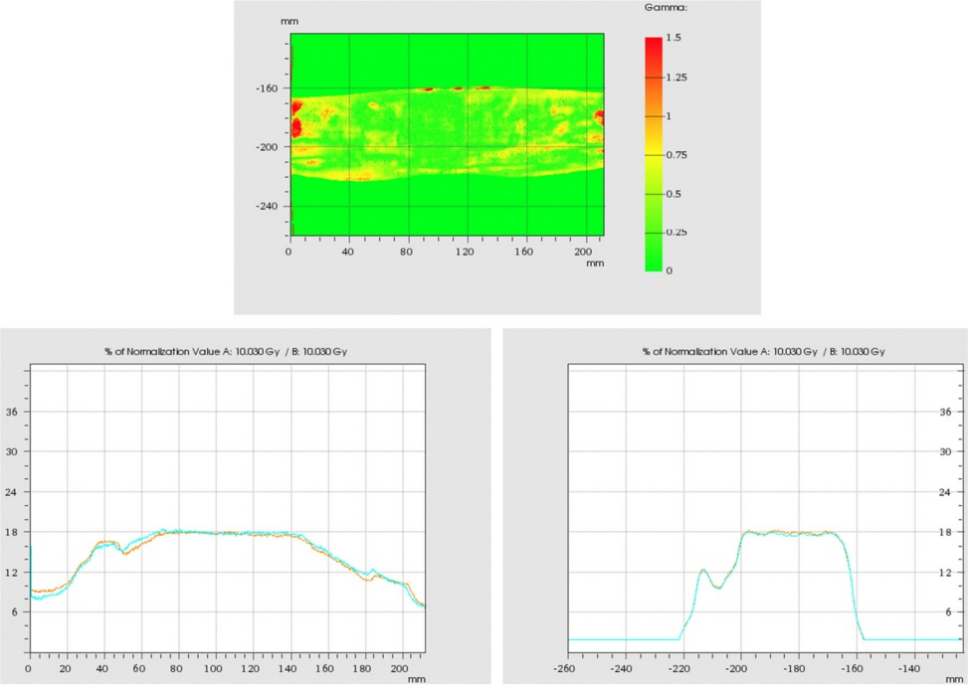
**Dosimetric verification of mARC vs. step-and-shoot original plan.** Top: Gamma distribution of GafChromic film measurement of the converted mARC plan (version c), compared with the original step-and-shoot plan. Pass criteria are 5% deviation in local dose and 3 mm distance to agreement, which are satisfied by 97.3% of measurement points. Bottom left: Example left-right profile, bottom right: target-gantry profile measured in both films (orange line: original step-and-shoot plan, blue line: mARC converted plan).

Planar dose distributions of both plan varieties (step-and-shoot vs. mARC) were measured in an acrylic phantom using GafChromic film. The agreement between both treatment modalities is excellent; an example is shown in Figure 7 for prostate plan version c – this is the version with least hybrid fields and most arclets, for which the difference between both plans is maximum. For this plan, 97.3% of the points pass the criteria of maximum 5% deviation in dose and 3 mm distance to agreement.

A comparison of the 3D-dose distribution of the step-and-shoot plans with the converted plans was performed using the PTW Octavius phantom with 729 2D-Array. For all measurements, between 93% and 100% of the points satisfied the pass criteria (again 5% deviation in dose and 3 mm distance to agreement).

**Figure 8:**
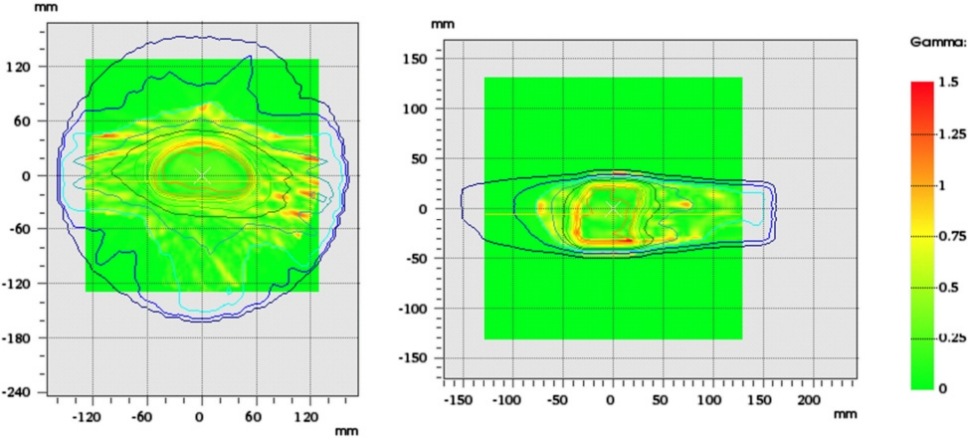
**Dosimetric verification of mARC vs. treatment planning system.** Gamma distribution of Octavius 729 2D-Array measurement of the converted mARC plan (version c), compared with the dose distribution exported from the Pinnacle TPS. One transversal and one sagittal slice are shown. Pass criteria are 5% deviation in local dose and 3 mm distance to agreement, which are satisfied by over 95% of measurement points in all slices.

In principle, validation of the treatment planning system is already proven by the fact that dosimetric verification for IMRT plans was checked in the commissioning phase [9] and in routine quality assurance, and since the deviation of the mARC plans from the step-and-shoot plans is negligible. However, we demonstrate these measurements for completeness. Besides, in the clinical routine it is more practical to perform routine mARC verification measurements directly (mARC plan vs. planning system) rather than the two-step approach of verifying the IMRT dose distribution and then comparing this with the converted plan. Verification of the mARC dose distribution before treatment is carried out using the PTW Octavius phantom with 729 2D-Array, which measured the irradiated mARC three-dimensional dose distribution with the treatment planning system. For all plans, over 95% of the points pass the criteria of 5% deviation in local dose and 3 mm distance-to-agreement (example shown in Figure 8), which provides an independent verification of the dose distribution of the mARC plan.

Treatment times were measured for all plans, an overview is given in Table 3. At our institution, standard prostate PTV plans take between 5 and 8 minutes to irradiate, whereas mARC plans take about 5 minutes for the flat 6 MV beam (with a maximum dose rate of 300 MU/min) and around 3 minutes for the flattening-filter-free 7 MV beam (using a maximum dose rate of 2000 MU/min). Even for the original IMRT plan with 19 beam directions, which was not adapted to a near-rotational setting, the treatment time was noticeably reduced by conversion to mARC (8:40 min to 5:15 min for 6 MV and 7:20 min to 3:35 min for FFF 7 MV, respectively).

**Table 3:** Treatment time comparison

**Prostate patient**		
Standard IMRT plans at our institution (6 MV flat)	5-8 minutes	
Converted mARC plans	6 MV	FFF 7 MV
Version a	05:15	03:35
Version b	05:00	03:30
Version c	05:05	02:55
**Head-and-neck patient**		
Standard IMRT plans at our institution (6 MV flat)	7-11 minutes	
Converted mARC plans	6 MV	FFF 7 MV
Version a	04:45	04:50
Version b	05:45	05:20
Version c	08:20	07:35

Treatment time (in minutes) with the different versions converted mARC for 6 MV and FFF 7 MV energies, compared with times usually observed at our institution for IMRT plans.

Depending on the number of gantry directions and amount of segments, standard head and neck IMRT plans usually require between 7 and 11 minutes radiation time at our institution. The converted plans lie within the range of 5 to 8 minutes, depending on energy and beam configuration. Clearly, a trade-off exists between treatment time and plan quality. The best dose distributions were found for the double arc treatment, which takes longest. The version c plans do not present a large advantage over standard plans from the point of view of treatment times (7–8 minutes), but have a very good dose distribution; if the conditions on the dose distribution are relaxed, faster treatment (4–5 minutes) becomes possible. This is a medical decision faced both for arc and IMRT treatment; in all cases, some time can be saved by switching from step-and-shoot plans to mARC.

## Discussion

We have explained a conversion algorithm which operates on any step-and-shoot IMRT plan to output a converted mARC plan. Even though only two patient examples are shown for a proof of principle, we have applied this technique in the clinic for a number of prostate patients. For all these patients, the 3D dose distribution was examined in detail before treatment, and with negligible dosimetric deviation similar to those presented in this manuscript. The implementation presented here offers the practical advantage that it can be applied to all treatment planning systems capable of IMRT optimization and DICOM export, which evidently expands the applicability of the mARC technique to a large range of Siemens customers who rely on TPS other than Prowess.

While in general every TPS can thus be used for the creation of mARC plans, it must be mentioned as a precaution that not every TPS is easily capable of achieving good optimization results for a large number of beams (compare [10]). It is not trivial to achieve good plans for all kinds of target structures and beam choices. For example, Pinnacle will more easily create good head and neck plans with 11 beams and 50 segments than using 36 beams with 36 (or even 50 segments), so special care must be taken in the choice of objectives to ensure good plan quality. In fact, it is well known in arc treatments that for complicated target volumes, one arc may not be enough, but two or three non-coplanar arcs are needed (e.g., [11,12]). The demands posed by a scenario as this may exceed the optimization capabilities of many non-arc-oriented TPS. However, simpler arc treatments can more easily be implemented and already provide large relief to both the patients and clinical schedule. Furthermore, the mARC conversion can be applied not only to create perfect mARC plans, but provides an intermediate planning option – a “poor man’s hybrid arc“ similar to our example using 19 beams. These plans can be optimized adequately by most IMRT-planning TPS; after conversion into “mARC“ their treatment time will be noticeably improved. For Pinnacle, we have obtained sufficiently good quality prostate plans with 30 beam angles, and high-quality head-and-neck plans with 72 segments from 36 directions, converted into two coplanar arcs. These plans result in stable and fast mARC treatment. For the creation of good-quality IMRT plans, it might also be tested to first split beams with two or more segments as demonstrated for prostate plan versions b and the head-and-neck plan versions a and c, and then re-optimizing again after the splitting, similar to the VMAT-approach described by [9].

An additional practical advantage of our implementation is that a step-and-shoot back-up plan is created for all mARC treatments (simply the original plan). At our institution, there are three matched linacs with 6 MV energy, but only one with mARC facility. In case of malfunction of the mARC linac, the mARC plan need not be recomputed and re-optimized as a step-and-shoot alternative version, but the original plan can be simply shifted to another linac, with no change in applied dose.

In implementations similar to the Pinnacle planning system, where IMRT is performed with a maximum number of segments distributed over all beam angles, the method offers the possibility to investigate beam-angle-optimization, which is a non-trivial problem in modern IMRT planning (for some recent approaches to this problem, compare, e.g., [13-15]). In our approach, the directions for hybrid arcs need not be pre-defined by the user, but are determined by the inversion algorithm in a way that the TPS assigns segments to those beam directions where they are “most needed”.

## Conclusions

We present an in-house conversion algorithm for converting IMRT step-and-shoot plans from any treatment planning system (Philips Pinnacle and Prowess Panther, in our example) into mARC plans. Creation of good quality IMRT plans using a large number of beams with few segments requires particular attention depending on the tumor location, but can be achieved by good design of beam geometry and inversion objectives, as is demonstrated for a clinically treated example. The converted mARC plans can be irradiated by the Siemens Artiste with no difference to “real“ mARC plans. The feasibility and dosimetric equivalence is demonstrated for the example of a prostate and a head-and-neck patient, using sets of different plans.

## Competing interests

The authors declare that they have no competing interests.

## Authors’ contributions

NL conceived the idea and general principle of the conversion algorithm. YD programmed the algorithm, implemented it in practice, performed all planning and plan comparison, and drafted the manuscript. Both YD and FN carried out the dosimetric verification measurements and discussed all issues related to the planning techniques and manuscript structure. All authors participated in the design of the study and read and approved the final manuscript.
